# Enhancing Handover for 5G mmWave Mobile Networks Using Jump Markov Linear System and Deep Reinforcement Learning

**DOI:** 10.3390/s22030746

**Published:** 2022-01-19

**Authors:** Masoto Chiputa, Minglong Zhang, G. G. Md. Nawaz Ali, Peter Han Joo Chong, Hakilo Sabit, Arun Kumar, Hui Li

**Affiliations:** 1 Department of Electrical and Electronic Engineering, Auckland University of Technology, Auckland 1010, New Zealand; masoto.chiputa@aut.ac.nz (M.C.); mizhang@aut.ac.nz (M.Z.); hakilo.sabit@aut.ac.nz (H.S.); 2 Department of Computer Science and Information Systems, Bradley University, Peoria, IL 61625, USA; nali@fsmail.bradley.edu; 3 Department of Computer Science & Engineering, National Institute of Technology, Rourkela 769008, Odisha, India; kumararun@nitrkl.ac.in; 4 Shenzhen Graduate School, Peking University, Shenzhen 518055, China; lih64@pku.edu.cn

**Keywords:** millimeter bands, Fifth Generation, handover, deep reinforcement learning, jump Markov linear system

## Abstract

The Fifth Generation (5G) mobile networks use millimeter waves (mmWaves) to offer gigabit data rates. However, unlike microwaves, mmWave links are prone to user and topographic dynamics. They easily get blocked and end up forming irregular cell patterns for 5G. This in turn causes too early, too late, or wrong handoffs (HOs). To mitigate HO challenges, sustain connectivity, and avert unnecessary HO, we propose an HO scheme based on a jump Markov linear system (JMLS) and deep reinforcement learning (DRL). JMLS is widely known to account for abrupt changes in system dynamics. DRL likewise emerges as an artificial intelligence technique for learning highly dimensional and time-varying behaviors. We combine the two techniques to account for time-varying, abrupt, and irregular changes in mmWave link behavior by predicting likely deterioration patterns of target links. The prediction is optimized by meta training techniques that also reduce training sample size. Thus, the JMLS–DRL platform formulates intelligent and versatile HO policies for 5G. When compared to a signal and interference noise ratio (SINR) and DRL-based HO scheme, our HO scheme becomes more reliable in selecting reliable target links. In particular, our proposed scheme is able to reduce wasteful HO to less than 5% within 200 training episodes compared to the DRL-based HO scheme that needs more than 200 training episodes to get to less than 5%. It supports longer dew time between HOs and high sum rates by ably averting unnecessary HOs with almost half the HOs compared to a DRL-based HO scheme.

## 1. Introduction

Fifth Generation (5G) mobile users need uninterrupted connectivity while consuming large amounts of data and media content when commuting [1]. Millimeter wave (mmWave) bands (i.e., 30−300 GHz on the radio spectrum) hold great potential, enabling 5G mobile users to experience gigabit rates and networks to meet traffic demands. However, a caveat to this is that mmWave communication is very susceptible to topographic and user dynamics. Common materials such as concrete, water, and even human bodies/movements [2] severely alter its cell patterns and ultimately its performance. This level of vulnerability in mmWave bands severely impacts mobility management in 5G mobile networks. To reduce that impact, research on efficient mobility management in 5G mmWave communication continues to gain momentum. In the recent past, 5G mobility management has been explored with machine and artificial intelligence (AI) learning solutions. Some of these include deep and reinforcement learning (RL) handoffs (HOs). The challenge is that most of the previous HO works [3,4,5,6] selected target cells on the basis of initial maximum network performance values. However, the challenge is that the optimum initial value does not always guarantee reliability of the connection after HO. For instance, the selection of mmWave target links based on the highest SINR values [4,5,6,7] does not always reveal the reliability of the link after a HO or caching [7] event. In most cases, HOs end up getting executed too early, too late, wrongly, or wastefully. Poor HOs negatively affect the selection of caching points [7] in edge computing too. To that effect, 5G mobile network performance is punctuated with gradual and abrupt changes. To reduce inconsistences in network performance, selection of the best target links requires understanding not just the immediate behavior after HO but also the long-term behavior.

To that effect, we propose an HO scheme that learns not just the immediate behavior of target links but also the likely behavior/pattern post HO. In this regard, we learn to predict the deterioration patterns of potential target links post HO. We use the jump Markov linear system (JMLS) [8,9] and deep reinforcement learning (DRL) to learn the feasible optimal deterioration pattern that chosen target links must adhere to for them to avoid wasteful HO. JMLS is known to account for abrupt changes [8] in system dynamics. We exploit this capability to predict the likely receivable power deterioration pattern of target links at the user. We strategically update the initial JMLS deterioration pattern with online DRL and meta training techniques. Meta training is a technique that reuses similar past training data to make new decisions. This reduces the request for new training datasets when making new decisions in a novel location. At HO, the predicted deterioration pattern of a target link is then compared against an optimal global desired deterioration pattern to understand the reliability of a target link and select the most stable one.

### 1.1. Related Works

The surging role/potential of mmWave bands in mobile networks such as 5G/beyond cannot be ignored. However, this also applies to its challenges, particularly in the mobility management support of 5G networks. The authors in for [10] instance claimed that higher propagation losses inherent in mmWaves must be addressed to sustain connectivity especially at ranges beyond 100 m and in non-line-of-sight (NLOS) settings. The authors in took four approaches to tackle the crucial problem of distance limitation owing to high spreading loss and molecular absorption that often limit the mmWave transmission distance and coverage range. These were a physical layer distance-aware design, ultra-massive MIMO communication, reflect arrays, and intelligent surfaces. These methods use machine and artificial intelligence (AI) learning for 5G. Various author between [11,12,13,14,15,16,17,18,19,20,21,22,23,24,25,26,27,28,29,30,31] suggested a move from centralized (used in most 4G systems) to decentralized mobility management algorithms using DRL. DRL in 5G ably learns and builds knowledge about different dynamics of mmWave channels [11,12,13,14,15,16,17,18]. For instance, by interacting with environment data, the authors utilized DRL to observe the available resource at network edges and provide a resource allocation scheme. This enhances user mobility management at the edge given user mobility context, transitions, and signaling exchange [11,12,13,14,15,16,17,18,19,20,21,22,23,24,25,26].

Exploiting various actor-critic different DRLs in [11,12,13,14,15,16,17,18,19,20,21,22,23,24,25,26,27], the authors e.g., in [18] proposed to jointly solve offload and resource allocation problems in 5G networks. The authors in [12] used a deep Q-learning-based task offloading scheme to select optimal BSs for users and maximize task offloading utility. In [13], Q-learning was integrated with the mobility robustness optimization (MRO) scheme and mobility-load-balancing (MLB) scheme to tackle traffic load and speed effects in 5G. In [29], a paradigm shift for leveraging time-consecutive camera images in handover decision problems was presented. DRL was used for deciding the handover timings. In [30], a DRL-based approach to solving the problem of joint server selection, task offloading, and handover in a multi-access edge computing (MEC) wireless network was proposed. On the other hand, in [31], HO and the power allocation problem in a two-tier HetNet, consisting of a macro base station and mmWave small base stations, were explored. The author developed a multi-agent reinforcement learning (MARL) algorithm based on the proximal policy optimization (PPO) method, by introducing centralized training with a decentralized execution framework. However, in all these schemes, highly mobile and dynamic users were hardly considered. Additionally, DRL requires thousands of samples to gradually learn useful policies [15]. Furthermore, DRL becomes terribly unstable/stochastic when learning systems with large local variances [16]. Thus, to guarantee continuous connectivity for 5G mobility, i.e., by not just satisfying channel input/state bounds but also considering abrupt and continuous disturbances, control approaches using Markov systems have been proposed in the literature. For instance, the authors in [20] used JMLS with expected maximization (EM) to predict abrupt deterioration behavior. Predictions were then enhanced using Viterbi algorithms. The Viterbi algorithm, however, requires accurate channel state information (CSI) to converge. In such cases, the authors in [26] argued that inaccurate training gradually cripples the accuracy of predictions, particularly at low signal-to-noise ratios (SNRs). To that effect, it was combined with meta data training, making the Viterbi proposed approach more reliable and less dependable on the changing and accuracy of the data. In [18], to tackle a distributed decision-making scenario, the author extended the JMLS formulation into game theory. Similarly, the authors in [17] incorporated particle-filter-based RL in JMLS to predict a finite number of disturbances within a randomly chosen sample of trajectories. This allowed the scheme to track/adjust to time-varying conditions in real-time. It is worth reiterating that none of the mentioned works analyzed the deterioration pattern of mmWaves to make an HO decision or utilized multiple users with very different levels of impact on mmWave propagation characteristics; they were all designed to operate in a single frequency band or with one user type.

### 1.2. Contributions

- We propose to use the JMLS to model the deterioration behavior/patten of mmWave target links and the formulation of HO policies for 5G mmWave networks. Given JMLS’s ability to account for abrupt changes [7], we analyze the pattern and learn to predict the extent of abrupt performance changes in the chosen target mmWave links before HO.
- We use DRL to update and optimize JMLS deterioration pattern predictions and learning. To help reduce training samples and, thus, have ample time to track pattern changes of rapid-varying channels in real time, we propose using meta learning techniques. Meta learning is a technique that automatically reuses training data from related past tasks or neighbors to make a new decision. This reduces the need for a new CSI/training dataset to make new decisions.
- We use the Kaiser–Meyer–Olkin (KMO) [25] test to measure the expected divergence of target links from the optimum deterioration pattern post HO to know their reliability in advance.

### 1.3. Organization

The remainder of this paper is organized as follows: Section 2 describes the proposed framework and its operation. The section further describes the resource allocation and optimization problems; Section 3 present adoptions of the JMLS–DRL solution; Section 4 analyzes the simulation results; Section 5 provides the conclusion.

## 2. Proposed Framework

We propose to use the likely received power pattern supplemented with SINR values to determine the best mmWave target cell/link. We first learn to predict and then analyze the received power deterioration pattern for four different types of users with respect to mmWave BSs. The four types of users are cars, pedestrians, cyclers, and e-bikers. For each user type, prior to HO selection, the scheme learns the likely mmWave user received power deterioration pattern given the effects of speed, topography, and channel state. The best target link is one whose likely deterioration pattern with distance is gradual and follows the global deterioration pattern generated from aggregative data samples from multiple mmWave BSs. The received power deterioration pattern is modeled using JMLS. It models how likely received power will deteriorate for a user given the NLOS and distance effects on the mmWave channel. Thus, in the first instance, the model learns and determines the desired optimal received power deterioration patterns for different user types using expected maximization (EM) [9]. EM automatically infers missing values of the link deterioration pattern over some states. Even though EM is robust, dynamic channel changes are not anticipated [10]. The EM estimations are, thus, optimized using DRL and meta training techniques.

Meta learning is loosely defined as an automatic learning and adaptation mechanism that improves accuracy by typically acquiring training from related tasks/users. The scheme only requires new training samples when the prediction error is bigger than the assumed predicted threshold. At HO, we have two deterioration patterns to consider: a global deterioration pattern formulated with aggregative data from all mmWave BSs, and a current local deterioration pattern formulated using local/individual BS channel data. Owing to the large data variance analyzed, the global pattern is regarded to be more accurate.

Thus, at HO, KMO test index values are used to determine the similarity levels between the global and local deterioration pattern for target links whose SINR is above the threshold. The level of divergence between the target link’s deterioration behavior and global pattern determines how reliable the target link is post HO. This is vital because mmWave links have a tendency of deteriorating from excellent to very poor performance immediately after HO. Thus, understanding the long-term connectivity endurance post HO is paramount for a reliable connection.

### 2.1. Manhattan Grid Mobility Model

**Figure 1:**
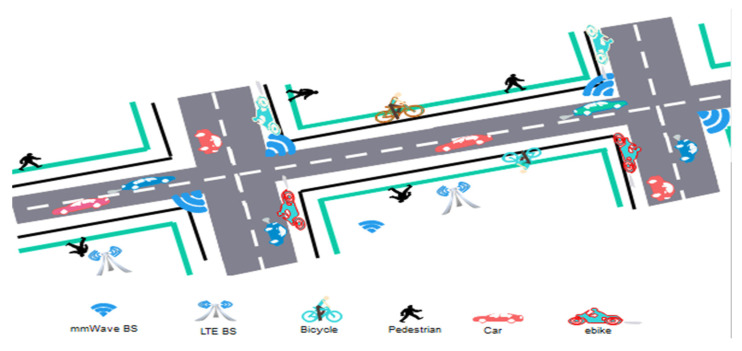
Multiuser type mobility model.

A Manhattan grid model is used to model the road network with streets and intersections (as shown in Figure 1) in an urban scenario. The road network area is 500 m × 100 m. We have four types of users, distributed evenly: pedestrians with speeds of 1.4 m/s, cyclers with speeds of 3–7 m/s, e-bikers with speeds of 8–9 m/s, and cars with speeds of 10–14 m/s. Cars within 3 m of each other adjust velocities every 3 s by 1–3 m/s to avert crashes. Each street consists of right and left lanes for each user type.

Given user directions, i.e., y={moving toward/away from a mmWave BS}, users traverse different streets. The probability of recovering the channel link just after being blocked ${\mathbb{P}}_{yr\;}$ and of remaining blocked ${\mathbb{P}}_{yb}$ is expressed as follows [12]:

(1a) $${\mathbb{P}}_{yr}=\frac{\;y}{K}\sum_{i=1}^{k}\frac{T^{r}}{T^{r}+T^{b}},$$

(1b) $${\mathbb{P}}_{yb}=\frac{\;y}{K}\sum_{i=1}^{k}\frac{T^{b}}{T^{r}+T^{b}},$$

where K  is the total number of samples, whist k∈K is the number of possible blockings; $T^{b}$ and $T^{r}$ are the mean nonblocking and blocking windows within a transmission range d. The rate of channel links switching from blocked to recovered and vice versa within d is 1/$T^{r}$ and 1/$T^{b}$, respectively. Accordingly, y is binary and assumed 1 when the users are moving toward a target BS. Otherwise, y is assumed to be 0, as the recovery of reconnection over the serving cell is minimal if user is moving away. The argument is that link recovery chances are high if a user is moving toward the direction of mmWave BS.

### 2.2. Outage Probability

Assuming that Θ is a set of optimization parameters for a given access policy π, the outage probability $P_{\pi }$ for the observable set of signals $Y_{k}\;$ can be defined as follows [2,11]:

(2a) $$P_{\pi }\left(Y_{k}|\Theta \right)\;≜P\left(\sum_{l}\sum_{s_{k}}b_{l}\log_{2}\left(1+\gamma_{t}\left(x\right)\right)\geq r^{m\zeta }\left({\hat{\gamma }}_{t}\right)\;\right),\;$$

where $\gamma_{t}$ and ${\hat{\gamma }}_{t}$ are the measured and target SINR, respectively, and $r^{m\zeta }$ is the targeted data rate given channel state $s_{t}\in S$. $b_{l}$ is the bandwidth for the given channel link l. We assume that all mmWave BSs directionally transmit equal maximum power P, and that all users have a receiver sensitivity of$\;x_{kmin}$. Thus, each serving mmWave BS (with either LOS or NLOS link) given, P, must satisfy the average received power of at least $\;x_{kmin}$. Moreover, given a threshold $\;x_{k0}$, where $x_{k0}>x_{kmin}$, any user–mmWave BS link that requires transmit power that exceeds P or does not meet $\;x_{k0}$ will not be established or lose connection, i.e., such a connection experiences a truncation outage at a given distance $d={\left(\frac{P}{\;x_{k0}}\right)}^{\frac{1}{\propto^{k_{L}}}}$ despite satisfying Equation (2). α is the path loss exponent in LOS and NLOS pathloss exponents [25]. Equally, given the cutoff threshold $x_{k0}$, LOS and NLOS users located at distances beyond ${\left(\frac{P}{\;x_{k0}}\right)}^{\frac{1}{\propto^{k_{L}}}}$ and ${\left(\frac{P}{\;x_{k0}}\right)}^{\frac{1}{\propto^{k_{NL}}}}$, respectively, from the target BS are unable to communicate owing to insufficient received power $x_{t}$. The data rate is defined as

(2b) $$r^{m}=b\;\log_{2}\begin{array}{c} \left(1+\frac{P{\left|h^{H}p\right|}^{2}}{\left(1+d^{\propto }\right)}\;F_{x}\left(\left|\theta_{k}^{l}\;\right|\right)\;\right) \end{array},$$

(2c) $$\varphi_{k}^{l}(\cdot )=\frac{1.4\times 10^{4}}{f_{c}(GHz)\cdot v(km/h)},$$

where $\theta_{k}^{l}=\frac{2d\sin\varphi_{k}^{l}}{\lambda }$ is the normalized central angle of arrival for beam *p*, v is user velocity under 50 km/h, $f_{c}$ is the carrier frequency.$\;{\left|h^{H}p\right|}^{2}$ is channel gain. and $F_{x}\left(\left|\theta_{k}^{l}\right|\right)$ denotes the Fejér kernel value. As user speed approaches zero and $F_{x}\left(\left|\theta_{k}^{l}\;\right|\right)\to 1$, SINR approaches the maximum. $F_{x}\;$approaches 0 as v increases [4].

### 2.3. Resource Allocation Problem

The minimum rate$\;{\mathbb{R}}^{m}$ requirement problem given outage and power constraints at d from a BS is defined as

(3a) $$\underset{\Theta }{\max}\underset{t}{\sum }\underset{S_{t},\;l}{\sum }\left(1-{\mathbb{P}}_{yb}\left(P_{\pi }^{m|x_{t}}+P_{\pi }^{m|u_{t}}\right)r_{l}^{m}\left(y\right)\right)\geq {\mathbb{R}}^{m},\;$$

where $P_{\pi }^{m|x_{t}}$ and $P_{\pi }^{m|u_{t}}$ are the LOS and NLOS conditional outage probability for a user in the m-th state, respectively. $r_{l}^{m}\;$is the maximum attainable data rate at user–BS distance d. The target receivable power$\;x_{t+1}$ at d needed to meet ${\mathbb{R}}^{m}$ in condition (2a) given outage constraints (1a)–(2b) is proposed in Equation (3b).

(3b) $$\;x_{t+1}=\max\underset{x_{t},u_{t}}{\sum }\left\{\frac{\hat{\gamma }}{\;\gamma^{min}}x_{t}-\frac{\alpha x_{t}{}^{2}}{\beta {\hat{\gamma }}^{2}\;}\right\},$$

where ${\left\{.\right\}}^{+}=\left\{max,\;0\right\}$.$\;x_{t}$ is the current received power in LOS. $\hat{\gamma }$ and $\gamma^{min}$ are the targeted and measured SINR needed to satisfy ${\mathbb{R}}^{m}$. It must be noted that, if there exists an infeasible SINR target in a certain user state, the resulting power demand, $x_{t+1}$, by users may diverge to infinity. This is due to each user link attempting to meet its own required SINR no matter how high the power consumption can be. Thus, α and β are power and SINR scaling factors, respectively, to substantially enhance reasonable deviations of $x_{t+1}$ in NLOS. The corresponding energy consumption for a given $x_{t+1}$ is as follows [24]:

(3c) $$\;E_{c}=\beta \left\{x_{t}\delta \frac{c\left(t-w\right)}{{\mathbb{R}}^{m}}+e_{0}*\zeta c\left(t-w\right)\right\},\;$$

where β denotes the price per unit energy consumption, c(t−w) denotes the actual number of packets received by the user at *t* during window *w*, $c\left(t-w\right)/{\mathbb{R}}^{m}$ is the latency, $x_{t}$ is the current received power at time *t*, $e_{0}$ is the unit energy per packet, and $e_{0}*\zeta c\left(t-w\right)$ denotes the energy lost due to lost packets (expected number minus the actual number of received packets) at *t* during window *w*. Given receivable $x_{t}$ and transmittable power P constraints (see Section 2.1), for optimum packet delivery latency, the maximum link utility problem is formulated as follows [19]:

(3d) $$\max\underset{P}{\sum }\underset{x_{t},}{\sum }\left\{x_{t+1}\delta c\left(t-w\right)-\zeta PE_{c}\right\},$$

where δ is the expected latency scaling factor given $x_{t+1}$ within *w.* $\zeta E_{c}$ is the latency discrepancy following a change from $x_{t}$ to $x_{t+1}$ as the user moves away from the serving BS. We learn to predict the long-term deterioration pattern $\left\{x_{t},\ldots ,x_{T}\right\}$ of the target links to ascertain its reliability in meeting the desired data rate prior to the next HO. We utilize JMLS properties to predict the likely gradual/abrupt deterioration behavior of target links [7].

## 3. JMLS System Definition

We first reformulate the resource allocation problem in Equations (3a)–(3d) into a JMLS learning form with system state, action, and reward defining the deterioration pattern.

### 3.1. The JMLS Representation

We propose the deterioration pattern learning algorithm and JMLS by describing Equations (3a–d) as follows:

(4a) $$\{\;\begin{array}{c} x_{t+1}=A(s_{t})x_{t}+B\left(s_{t}\right)u_{t}+w_{t}\;\\ y_{t}={𝓇}^{min}\left(s_{t}\right)x_{t+1}+v_{t},\;\\ \;ℳ=\left(\Theta ,\;P\left(S\right),\pi ,P_{\pi }\right)\; \end{array}\;\;$$

where $x_{t}\in X$ is the current received power in the LOS given state $s_{t},$ and $u_{t}\in U$ is the estimated received power discrepancy due to blockage/NLOS effects. It is related to $x_{t}$ by $u_{t}=-Kx_{t}$ where K is the control factor of the power and SINR scaling factor in Equation (3b); $A(s_{t})$ and $B(s_{t})$ are the SINR/power coefficient matrices in Equation (3b). $v_{t}\sim N\left(0,Q(s_{t})\right)\;$and $w_{t}\sim N\left(0,R(s_{t})\right)\;$are the data rate and received power measurement noise, respectively. Measurement noises are influenced by the competing effect of change in gain, angular and linear transmission distance, user speed, etc. for the same SINR requirements (see Equation (3a–c)). $s_{t}$ denotes a state governing for parameter set $\Theta =\left\{A,\;B,\;R,{𝓇}^{min},Q,P\left(S\right)\;\right\}$. $s_{t}$ belongs to a set of Markov stochastic decisions $ℳ=\left\{m_{1,}m_{2}{,\ldots ,m}_{M}\right\},\;$and $m_{M}\;$determines which state is active at time *t*.

(4b) $$s_{t}=\left\{v,r_{t},T_{t},d_{t},\eta_{t}\right\},\;$$

where $v=\left[v_{1},\ldots ,v_{T}\right]$ is a vector of user velocity, $r_{t}=\left[r_{1},\ldots ,r_{t}\right]$ is a vector of possible user data rate, $T_{t}=\left[t_{1}^{m},\ldots ,t_{N}^{m}\right]$ is a vector of average service time, $d=\left[d_{t}^{m},\ldots ,d_{T}^{m}\right]$ is a vector of transmission distances with the same SINR, and $\eta =\left[\;\eta_{1},\ldots ,\eta_{N}\right]$ is a vector of user direction in the *n*-th sample.

Following a transition to $x_{t+1}$, the immediate reward ${𝓇}^{min}\left(s_{t}\right)$ for the observed signal $y_{t}\in Y$ is defined as a function of energy efficiency.

(4c) $${𝓇}^{min}\left(s_{t}\right)=\frac{r^{m}\left(s_{t},a_{t}\right)}{P_{t}},\;$$

where $r^{m}\left(s_{t},a_{t}\right)\;$is a data rate greater than ${\mathbb{R}}^{m}$ in Equation (3a). The likely rate discrepancy between a user and mmWave BS is expressed as

(4d) $$Q(s_{t})=\delta_{k}\frac{\left(P_{t}-x_{t}\right)r^{m}\left(s_{t},a_{t}\right)}{P_{t}},$$

where $\delta_{k}$ is the scaling factor of the rate discrepancy for each state, *s* at time *t* given maximum rate $r^{m}\left(s_{t},a_{t}\right)$. The transition probability between states with $x_{t}$ and $x_{t+1}$ is

(4e) $$P\left(S\right)≜P\left(s_{t+1}=m_{j}|s_{t}=m_{i}\right).\;$$

Assuming that N samples from different mmWave BSs at time t are collected within each window w and arranged in ascending order of the users’ distance from serving BSs, the transmission energy cost function is defined as follows:

(4f) $$J\left(x_{t}\right)=E\left\{\sum_{j=1}^{N}\|x_{j}{\|}_{Q\left(s_{t}\right)}^{2}+\sum_{j=0}^{N-1}\|u_{t}{\|}_{R\left(s_{t}\right)}^{2}\right\}\;,$$

where first and second factors in Equation (4f) represent the sum-weighted norm energy cost for received packets and lost packets over $X_{N}=\left\{x_{0},\ldots \;x_{N}\right\}$, respectively. $J\left(x_{t}\right)\;≜\sum_{j=1}^{N}\;E_{c}$.

### 3.2. Initial Deterioration Path Training

**Figure 2:**
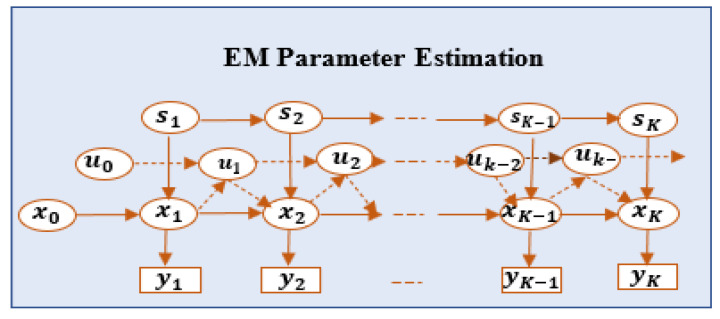
Dynamic Bayesian representation of JMLS composed of three variables and related deterioration variables over adjacent timesteps K.

**Figure 3:**
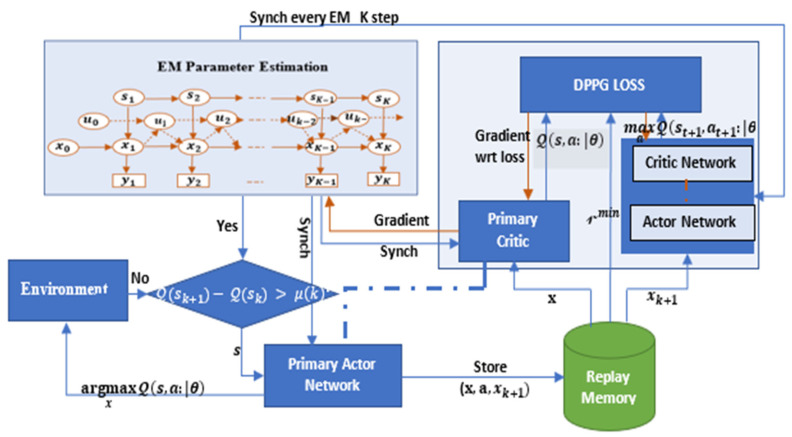
Deep deterministic policy gradient (DDPG) algorithm structure.

$Y_{T}$, $X_{T}$, and $S_{T}$ denote a sequence of observed data rates $\left\{y_{1},\;\ldots ,\;y_{T}\right\}$ over corresponding receivable power values $\left\{x_{0},\ldots \;x_{T}\right\}$ and $\left\{s_{1},\;\ldots ,\;s_{T}\right\}$ states until time T. The JMLS learning problem in each user type is to define the likely sequence $X_{T}\;$and parameter Θ that maximize the likelihood function $P\left(X_{T}|\Theta ,Y_{T}\right)$ given a finite observation in $Y_{T}$ over $S_{T}$ for all $k_{1}$, …, $k_{2}$ ∈ T at distance $k_{1}\leq k_{2}.\;$The initial deterioration pattern estimator upon which we design our framework for received power pattern *X* is the EM algorithm in [12]. EM uses Bayesian inference to automatically infer the optimal value set of Θ for $X_{T}$ [12] at each step k, as seen in Figure 2; the value function can be written as

(5a) $$\mathbb{Q}\left({\Theta |\Theta }^{k}\right)=E\left[\log P\left(X_{T},S_{T},Y_{T}|\Theta \right)|Y_{T},\;\Theta^{k}\right],$$

such that

(5b) $$\Theta^{k}=\arg\underset{x\;\in \;X}{max}\mathbb{Q}\left(\Theta |\Theta^{k}\right),$$

where Θ^(*k*)^ is the current parameter estimate at iteration k. The change, $\Delta Q(s_{t})$, between $s_{k}\;$and $s_{k+1}\;$states must satisfy condition (5c) to avoid abrupt changes or shocks in data rate.

(5c) $$\left|Q(s_{k+1})-\;Q(s_{k})\right|<\mu {\left(k\right)}^{v}\;,$$

where $\mu {\left(k\right)}^{v}\;$is the averaged data rate discrepancy between states $s_{k}$ and $s_{k+1}$ for a user with velocity *v*. In Equation (5c), a smaller difference denotes a lower change between $x_{k}$ and $x_{k+1}$, defining deterioration pattern $X_{T}$. $Q(s_{k+1})\;\mathrm{and}\;Q(s_{k})$ can be chosen independently. Obtaining full or accurate CSI to determine the pattern may be difficult owing to rapid changes in mmWave channels. Furthermore, EM cannot handle such switching dynamics [12]. Thus, instead of recomputing the steps in Equations (5a–c) to refine pattern X, as more CSI about $Y_{T}$ is obtained, we use online DRL with EM estimations of X as the initial experience to determine user target data rates $Y_{T}$, as seen in Figure 3.

### 3.3. Deep Reinforcement Learning in EM-Estimates

As seen in Equation (5b), EM estimates the maximum obtainable received power x, i.e., the upper bound of desirable received power in each state needed to obtain a high SINR, $a\in A_{x},$ and, hence, data rate, y, efficiently. The role of DRL, given optimum maximum receivable power $-x^{*}$ per state, is to determine the minimum/lower bound of receivable power $x^{*}$ needed to obtain the same JMLS value, $a\in A_{x}$, efficiently about the same state. It must be both noted and emphasized that the power at the receiver can randomly vary with time, space, and frequency. This may trigger erroneous reception at the receiver. Rectifying or averting the errors may need a high transmit power (which is energy-inefficient and is beyond the limit) to meet the desirable receivable power and receive the same amount of user data within a given QoS/SINR requirement. However, if the gain of the channel is high in the peak, even if the received power is lower (e.g., in NLOS), this permit using lower receivable power to receive the same/similar amount of data while maintaining the same given QoS/SINR. Thus, knowing the pattern of noy only the maximum but also the minimum receivable power prior to the HO decision is vital. Hence, DRL is used to determine minimum desirable power given the maximum by EM estimation. Here, DRL uses EM data as the initial experience (meta data) to determine the least expected receivable power needed to give $a\in A_{x}$. In that case, the DRL agent has to consider only the SINR value, $a\in A_{x}$, possible for −x in EM and find the power x that gives the highest directly obtainable reward plus expected accumulated future reward of the resulting states s. The EM’s Q value for the (−x, a) pair is used as meta data by the agent to find the SINR that gives the smallest DRL Q value with a function value V. The optimal value function $V^{*}$ is obtained by solving $x_{ko}$ for each given $-x_{ko}$ in Figure 2.

(6a) $$V^{*}\left(x_{ko}\right)=\underset{\pi }{\max E}\left\{{𝓇}^{min}\left(-x^{*}{}_{t},a\left(s_{t}\right),x^{*}{}_{t}\right){|}_{s_{t},\pi \left(x_{t}|\theta \pi \right)}\right\}.$$

Technically, for a given optimum pattern $-X^{*}$ in Equation (5c), the algorithm uses corresponding optimized parameter sets θπ and policy $\pi \left(s_{t}|\theta \pi \right)$ as input to DRL. The DRL scheme then determines the minimum desirable value $x_{t}$ needed to achieve $a\left(s_{t}\right)$. It uses corresponding maximum value $-x_{t}$ determined by EM in each state $s_{t}\;$as the initial experience and improves it by minimizing the expected energy cost, $J\left(x_{t}\right)$. The policy $\left(s_{t}|\theta \pi \right)$ is defined as

(6b) $$\pi =\underset{J}{argm\mathrm{in}}\left\{Q\left(a_{t},x_{t}|\theta \pi \right)+\varepsilon \underset{s_{t}\in S}{\sum }P_{\pi }\left(x_{t}|-x_{t},a_{t}\right)J^{*}\left(x_{t}\right)\right\},\;$$

where $P_{\pi }\left(x_{t}|-x_{t},a_{t}\right)\to \;\left[0,\;1\right]$ denotes the probability of transition from $-x_{t}$ to $x_{t}$ without change, a∈A, with least possible energy cost $J^{*}\left(x_{t}\right),$ in $s_{t}$. The optimal policy π derives the smallest possible value of $Q\left(-x_{k},a_{k},x_{k}|\theta \pi \right)$; hence, $J^{*}\left(x_{t}\right)\;$in Equation (4f) satisfies the following Bellman equations:

(6c) $$J^{*}\left(x_{t}\right)=\mathrm{if}\;s^{*}\in S\vee x_{t},\;\mathrm{else}\;$$

(6d) $$J^{*}\left(x_{t}\right)≜\underset{x}{\min}E\left[{𝓇}^{min}\left(a\left(s_{t}\right),x_{t}^{*}\right)+\underset{x_{t}\in X}{\sum }P_{\pi }\left(x_{t}|-x_{t},a_{t}\right)J\left(x_{t}^{*}\;\right)\right],\;$$

where $s^{*}$ are goal states where condition (5c) is satisfied.

### 3.4. Deep Deterministic Policy Gradient (DDPG)

We use the deep deterministic policy gradient (DDPG) to improve the accuracy of the pattern. DDPG is combined with DQN on the premise of the EM algorithm in order to further enhance the stability and effectiveness of network training. This makes it more conducive to solving issues of continuous state and action space. Technically, DDPG uses DQN as the experience replay memory and the target network to solve the problem of nonconvergence to approximate the EM function values in neural networks. It is, thus, an actor-critic and model-free algorithm. It learns policies using highly dimensional observation and action spaces. In this respect, agents use three modules: primary network, target network, and replay memory.

Primary networks match actions (SINR ratios in JMLS parameter sets) with expected received power using a policy gradient method. It consists of two deep neural networks, namely, primary actor and primary critic neural networks. On the other hand, the target network sets target values $y_{t}$ for the optimal receivable power $x_{t}$ with pattern X given by EM estimations. The replay memory stores the tuple experience from EM Bayesian estimators and environment via the actor network given condition (5c). Experience tuples include the current and next state, the SINR ratio value following the transition between states, and the reward for choosing the received power level in $X_{T}$. Replay memory updates are randomly sampled for training the primary critic network and setting the target in the target network for the eventualities in Equation (5c).

Given EM parameter set θ and policy $\pi \left(s_{t}|\theta \pi \right),\;$the cost policy gradient $\nabla_{\theta \pi }J$ gives the values of $x_{t}\in X_{T}\forall \;y_{t}\;$with a minimum change in $\nabla_{\theta a}Q\left(a_{t},x_{t}|\theta \pi \right)$ between $-x_{t}$ and $x_{t}$, and the corresponding maximum change $\Delta {𝓇}^{min}\left(-x_{t},a_{t},x_{t},s_{t}\right)$ for each value$\;x_{t}\;$transitioning from $-x_{t}$ is defined as

(7a) $$\nabla_{\theta \pi }J\approx \underset{\pi }{\max}E\left[\Delta {𝓇}^{min}\left(a_{t},s_{t}\right){|}_{s_{t},\pi \left(x_{t}|\theta \pi \right)}\nabla_{\theta a}Q\left(a_{t},x_{t}|\theta \pi \right)\right].$$

The optimal value $J^{*}\left(x_{t}\right)$ gives the highest possible expected future reward and lowest discrepancy from target values for each state. The policy gradient is explored by the primary actor neural network, and the value function Q for the (x,a) pair is used by the agent to find the SINR ratio a and received power x that gives the lowest Q value and highest reward. Value iteration in DDGP terminates when ∀s ∈ S, $|J_{k}$(x) −$J_{k}$(−k)| ≤ ε, and termination is guaranteed for ε > 0. ε is similar to a greedy strategy with probability 1−ε [27]. Here, ε decays as more iterations (and, hence, more experience) are gained. The primary critic network updates θa by minimizing loss function Ls(θπ), which is defined as

(7b) $$Ls\left(\theta Q\right)=E\left({\hat{y}}_{t}\;-\;Q\left(a_{k},-x_{k}|\theta \pi \right)\right),$$

where ${\hat{y}}_{t}$ is the target network value and can be obtained by

(7c) $${\hat{y}}_{t}={𝓇}^{min}\left(a,x_{t}\right)+\varepsilon Q^{k}(x_{k},\pi^{k}\left(s_{k+1}|\theta_{\pi }^{T}\right)|\theta_{a}^{T}).$$

Here, $\varepsilon Q^{k}(x_{k},\pi^{k}\left(s_{t+1}|\theta_{\pi }^{T}\right)|\theta_{a}^{T})$ is obtained through the target network, i.e., the network with parameters θπ, from EM with −X values and θa from X generated over time for minimum desirable receivable power. The new values of Equation (5c), i.e., patterns, are updated by minimizing loss in Equation (7b). The gradient of Ls(θQ) over $X_{T}$ is calculated by its first derivative, which can be denoted as in [14].

(7d) $$\nabla_{\theta \pi }Ls\left(\theta Q\right)=E\left(2\left(y_{t}-Q\left(a,x_{t}|\theta \pi \right)\nabla_{\theta a}Q\left(a,s_{t}|\theta \pi \right)\right)\right).$$

According to Equation (7d), the parameter $\theta_{Q}$ of the primary critic neural network can be updated. Specifically, at each training step, a mini-batch experience $\langle \;s_{t},\;a_{t},R^{imm},\;s_{t+1}\rangle ,\;t\in \left\{1,\;.\;.\;.,\;k\right\}$ is randomly sampled from replay memory. For each point in $X_{K}$, the target network value is regarded as the previous and current version of EM parameters $\theta_{\pi }^{T}$and $\theta_{Q}^{T}$. At each iteration, $\theta_{\pi }^{T}$ and $\theta_{Q}^{T}$ in Equations (7c,d) are updated with a weighted combination of the previous state. The prediction of target path takes the form of a weighted combination of the following models:

(7e) $$\theta_{\pi }^{T}({\tilde{x}}_{k})=𝓌\theta_{\pi }(-{\tilde{x}}_{k})+\left(1-𝓌\right)\theta_{\pi }^{T}(-{\tilde{x}}_{k}),\;\theta_{Q}^{T}({\tilde{x}}_{k})=𝓌\theta_{Q}({\tilde{x}}_{k})+\left(1-𝓌\right)\theta_{Q}^{T}({\tilde{x}}_{k}),$$

where ω ∈ [0, 1] is the weight computed using a Gaussian kernel parameterized by the transmission distance metric $d_{k}\in \;{\tilde{s}}_{k}$.

(7f) $${𝓌}_{k}=\exp\left(-0.5{\left(x-\mu_{k}\right)}^{T}d_{k}\left(x-\mu_{k}\right)\right).$$

Target neural networks generate target or ideal values for training and reoptimizing the deterioration pattern $X_{T}$ from $-X_{T}$ on the basis of EM and replay updates. Thus, EM estimations in each iteration are used as meta data for DDPG. The target neural network has a similar network structure to the primary network, i.e., similar neural network structure and initialization parameters. In the training process, the parameters of the target actor and critic networks are updated slowly (soft replace) by EM estimated values. Here, instead of directly and randomly training parameters of the primary actor and critic networks to further enhance the stability of the training process, we copy EM estimations as ideal initial values. Replay memory stores EM experience tuples, thus formulating $X_{T}$, and each value update $x_{t}\in X_{T}$ includes a tuple $\langle -x_{t},\;a_{t},R^{imm},\;x_{t}\rangle$ update.

Figure 3 shows the structure of the proposed JMLS–DDPG algorithm. The DDPG algorithm takes the EM parameter dataset and maximum receivable power values −X as initial input to determine the minimum receivable power values of a pattern. Given that the power effects on SINR can be reduced in high-channel-gain locations, afterward, the DDPG agents output the minimum receivable power values X needed to maintain the same SINR ratio previously predicted and set by EM estimations for $S_{K}$. The corresponding reward of $x_{k}$ in EM is copied, and the SINR that is beneficial to the agent to achieve the goal gives a positive reward; on the contrary, it gives a negative reward if condition (5c) is not fulfilled. The current state information, the SINR ratio, the reward, and the state information of the next minimum desirable receivable power are stored in the replay pool. Meanwhile, the neural network trains the experience and continuously adjusts the SINR strategy by randomly extracting sample data from the EM pool, and it uses the gradient descent approach to update and iterate network parameters, so as to further enhance the stability of pattern X and the accuracy of the algorithm. Using EM experiences as initial training data input to DDPG restricts the search range for optimal minimum receivable power values. Thus, any observed mmWave BS data rate not meeting the corresponding receivable power is immediately discarded for training or consideration. This in itself technically reduces the training sample for DRL and, hence, convergence time. Ultimately, the improved DRL HO is obtained by combining DDPG with EM predictions acting as a meta training sample. Finally, the pattern model is integrated into the HO platform for HOs.

### 3.5. Online Update of Target Deterioration Path

**Figure 4:**
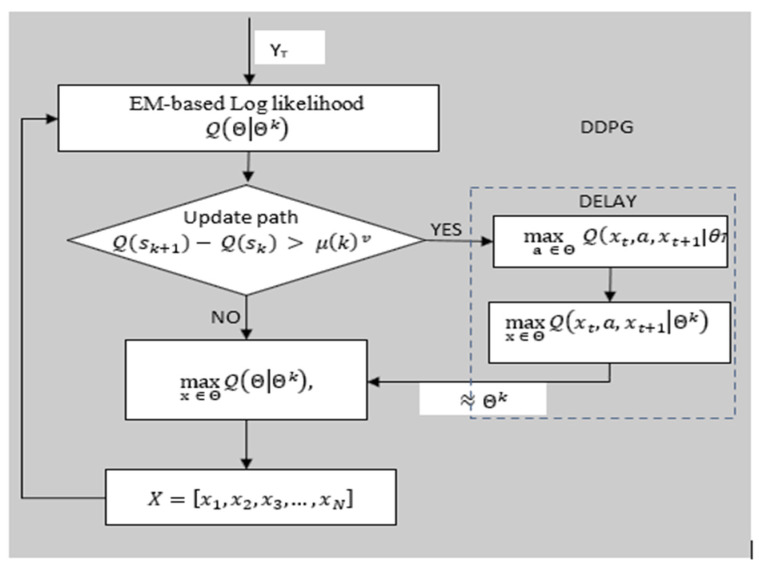
Workflow of JMLS–DDPG algorithm.

DDPG subdivides the training network structure into an online network and target network (see Figure 3). The online network is used to output the minimum expected received power in real time, evaluate SINR ratio values, and update network parameters through online training, which includes the online (primary) actor network and online critic network. The target network includes the target actor network and target critic network, which get updated by EM values. The target actor network system, however, does not carry out online training. For each user type, the estimated path $X_{N}$ is only re-estimated from new training samples when the pattern prediction error based on EM estimates is too much larger than the minimal desired received power pattern. It, therefore, follows that, when the error given the energy efficiency is small enough such that the channel gain compensates for the power loss to maintain the desired SINR, the corresponding EM information used to generate received power pattern $X_{t}$ is regarded to provide reliable training sample for the target network in DDPG. EM data are, thus, re-encoded to generate new training samples for the DRL and to set new targets over$\;{\tilde{S}}_{t}$, a process henceforth referred to as meta-training. If indeed the pattern of link deterioration is successfully followed by the target mmWave network, then ${\tilde{X}}_{t}\;$represents the true channel link deterioration behavior from which $Y_{t}$ is obtained. Consequently, the corresponding pair ${\tilde{S}}_{t}$ and $Y_{t}$ parameter set $\theta_{\pi }(s_{t})$ can continue being used to retrain DDPG instead of requesting new CSI from the environment in Figure 3. The model can be efficiently and quickly retrained with a relatively small number of new training samples. A natural drawback of decision-directed approaches such as the Bayesian in EM is their sensitivity to decision errors. For example, if the link fails to successfully sustain connectivity, then the meta training samples $-\tilde{X}$ of $\tilde{X}$ over ${\tilde{S}}_{t}$ do not accurately represent the channel behavior results in $Y_{t}$. In such cases, the inaccurate training sequence may gradually deteriorate the accuracy of DDPG predictions, making the proposed approach unreliable, particularly in low-SINR areas where link deterioration pattern errors occur frequently. Nonetheless, when pattern errors are less frequent in EM, the effects of decision estimate errors of ε, i.e., the number of errors in a pattern, can be used to decide when to generate meta training. For instance, we retrain with new training samples in DDPG only when the number of errors is larger than some threshold. Using this approach, only accurate meta training data are used, and the effect of decision errors is controlled. When using new training samples, we cleverly focus attention on states with non-converged pattern values, i.e., where Equation (5c) is not fulfilled. Our online training mechanism is summarized in Algorithm 1. The Workflow in Figure 4 summarizes the steps in Algorithm 1. In particular, EM estimates the initial receivable power pattern. If, however, the date rate discrepancy condition is not satisfied in Equation (5c), DDPG in Figure 4 is conditionally evoked to improve the prediction of the target link deterioration pattern when EM fails to meet the data rate condition in Equation (5c). DDPG, as earlier alluded to, cleverly uses the maximum SINR to find the minimum expected receivable power of each state defining the deterioration pattern.
**Algorithm 1:** JMLS–DRL-Based Pattern Algorithm.**Input:** User mobility model parameters, ${\mathbb{P}}_{y}$. v Parameters about DC communication: transmission power limits, bandwidth, channel gain, and NLOS and LOS path loss exponent. Observed states S; Set of observed signals $Y=\left[y_{1},y_{2},y_{3},\ldots ,y_{N}\right]\in \mathbb{R},\;$ **Output**: mmWave deterioration path $\;X=\left[x_{1},x_{2},x_{3},\ldots ,x_{N}\right]$ for target linkInitialize the deterioration path estimations**for** t=1 **do**  Draw $y_{t}$ for JMLS parameter estimation Θ, where $\left(X_{T},S_{T},Y_{T}|\Theta \right)$**Estimate Maximization (EM):**    $Q\left(\Theta |\Theta^{k}\right)=E\left[\log P\left(X_{T},S_{T},Y_{T}|\Theta \right)|Y_{T},\Theta^{k}\right],$  $\Theta^{k}=\arg\underset{x\;\;\in \;X}{max}Q\left(\Theta |\Theta^{k}\right)\;$Define pattern: $X=\left[x_{1},x_{2},x_{3},\ldots ,x_{N}\right]$  **for** $x_{N}$ **do**
    **if** $Q(s_{k+1})-Q(s_{k})>\mu {\left(k\right)}^{v}\;$**then**      **update** $x_{N}$ **with DRL**    **else**      repeat step 6 for all  X    **end if**  **end for****Update EM deterioration path estimations with DPPG**Re-estimate $Q(s_{k+1})$ using primary network $Q\left(s,a|\theta_{\pi }\right)$Initialize target network parameters with EM parameter setInitialize replay memory using EM samples. **for** each EM step **do**     Observe user state $s_{t}$ and SINR ratio $a_{t}\in \theta_{\pi }$     Execute $a_{t}\in \theta_{\pi }$ and state $x_{t}$    Observe change in ${𝓇}^{min}\left(a_{t},s_{t}\right)$ and $Q\left(s,a|\theta_{\pi }\right)\;$    Update EM tuple < $s_{t}$,$a_{t},{𝓇}^{min}$, $s_{t+1}$ > in replay memory.Compute target value ${\hat{y}}_{t},$ update $Q\left(s,a|\theta_{\pi }\right)$ and minimizing lossUpdate target neural networksUpdate EM with $\theta_{\pi }$ and recompute steps 6–12 for all X      **end for**    **end for****end for**

### 3.6. Global Path and Local Path Optimization Formulation

The local pattern is formulated on the basis of local CSI from one mmWave BS. The local agent, thus, considers only the SINR ratio $a\in A_{x}$ and corresponding received power x values possible in the local environment over given states ${\tilde{S}}_{t}$. The long-term function for the local deterioration pattern is expressed as

(8a) $$Q_{LP}\left(a_{t},x_{t}|\theta \pi \right)≜E\left[\overset{T}{\underset{t=0}{\sum }}\delta^{t}\left\{{𝓇}^{min}\left(a_{t},x_{t}\right)+\varepsilon Q(x_{t+1},\pi \left(x_{t+1}|\theta \pi )\right)\right\}\right],$$

where *δ* ∈ (0,1) is the discount factor and approaches 1 with more training samples. The global deterioration pattern is formulated on the basis of collective SINR ratio $a_{t}$ and received power $x_{t}$ values from different mmWave BSs over ${\tilde{S}}_{t}$. The value function $Q_{GP}$ is

(8b) $$Q_{GP}\left(a_{t},x_{t}|\theta \pi \right)≜\underset{a\in A_{x_{k}}}{\sum }P_{\pi }\left(a_{t}|x_{t}\right)*\frac{\alpha }{K}\left\{Q\left(x_{t+1}a_{t},x_{t}|\theta \pi \right){𝓇}^{min}\left(x_{t+1},a_{t},x_{t}\right)\right\},\;$$

where $P_{\pi }\left(a_{t}|x_{t}\right)$ is the probability of receiving x given α in state s by EM; α is the learning rate over K samples in EM.

### 3.7. Handoff Considerations

We use the Kaiser–Meyer–Olkin (KMO) test [25] to test how much each individual/local mmWave target link’s expected deterioration pattern, given the user speed, deviated from its optimized global deterioration pattern. The global deterioration pattern is formulated by collecting training sample from all mmWave BS with respect to user type/speed just like the complete report table (CRT) in [4]. The local deterioration pattern is based on data gathered from an individual BS’s local environment with respect to a user’s type. It is similar to the report table (RT) user data in [4]. Given all the target BSs with at least 3 dB SINR above the threshold, the KMO indexing test is used to find the level of correlation between an optimized global deterioration pattern and that of a target link at the time of the HO request. The KMO overall index value correlation is defined as follows:

(9a) $${\mathrm{KMO}}_{\hat{x}}=\frac{\sum_{x\neq \hat{x}}R_{x\hat{x}}^{2}}{\sum_{x\neq \hat{x}}R_{\hat{x\hat{x}}}^{2}+\sum_{x\neq \hat{x}}a_{x\hat{x}}^{2}},\;$$

where $R=\left[r_{xd}\right]$ is the correlation matrix, and $A=\left[a_{xd}\right]$ is the partial covariance matrix, where $a_{xd}$ is defined as

(9b) $$a_{x\neq \hat{x}.m}=\frac{r_{x\hat{x}}-r_{x.m}r_{\hat{x}.m}}{\left(1-r_{xm}^{2}\right)\left(1-r_{\hat{x}m}^{2}\right)},\;$$

and

(9c) $$\;r_{x\hat{x}}=\frac{\sum_{t=0}^{T}\left(x_{t}-{\hat{x}}_{t}\right)\left(d_{t}-{\hat{d}}_{t}\right)}{\sqrt{\sum_{t=0}^{T}{\left(x_{t}-{\hat{x}}_{t}\right)}^{2}\sum_{t=0}^{T}{\left(d_{t}-{\hat{d}}_{t}\right)}^{2}}},\;$$

where $x_{t}\in X_{T}\;$is the optimum lower bound target link value of received power at state $s_{t}$. $d_{t}\in s_{t}$ is the minimum expected user–BS link distance, and ${\hat{x}}_{t}$ and $\;{\hat{d}}_{t}$ are values for the global deterioration path. The KMO test takes values between 0 and 1, as summarized in Table 1. The general rule for interpreting measurements is provided in Table 1. In this study, we selected the target cells with a KMO index of 0.751. If the KMO index value is less than 0.7, the target link is most likely not suitable for HO consideration although it might have the highest initial SINR. Additionally, during the HO phase, if the serving BS still has a SINR value of 3 dB, the user maintains the connection to the serving gNB. This avoids wasteful HOs. Otherwise, we execute the HO process and then go back to prediction phase.

### 3.8. Measurement Definition

We measured the number of repeated HOs to ascertain if the HO scheme can reduce the number of the wasteful HOs. Repeated HOs mean that the HO scheme is reselecting the same serving BS in which the user is already connected to for another HO. This is wasteful because there is no need to reselect the same BS for HO but rather maintain the link. We also analyzed the sum data rate of mmWave BSs using different HO schemes. Additionally, we analyzed the HO overhead for different schemes. The principle is that a higher overhead reflects a more wasteful HO scheme with the bandwidth. Lastly, we analyzed the performance of our proposed scheme compared to another scheme, dubbed the DDPG only scheme. The DDPG only scheme does not use the meta training technique and does not consider condition (5c). Specifically, it uses random training samples rather than EM refined samples. We also analyzed performance compared to the existing soft HO DC model HO scheme in [3]. This scheme only selects the best target cell by averaging the SINR/data rate.

## 4. Simulation Results

**Figure 5:**
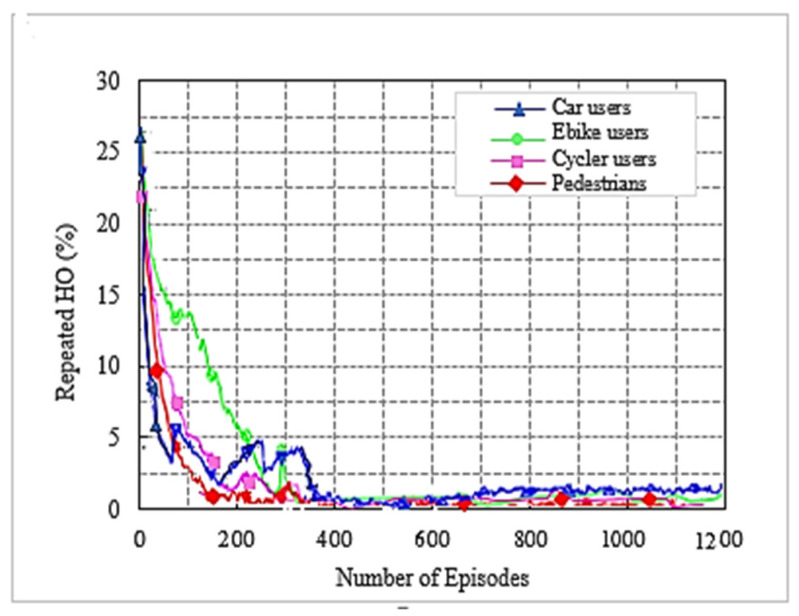
Number of wasteful HO as a function of the number of training episodes for DDPG only HO scheme.

**Figure 6:**
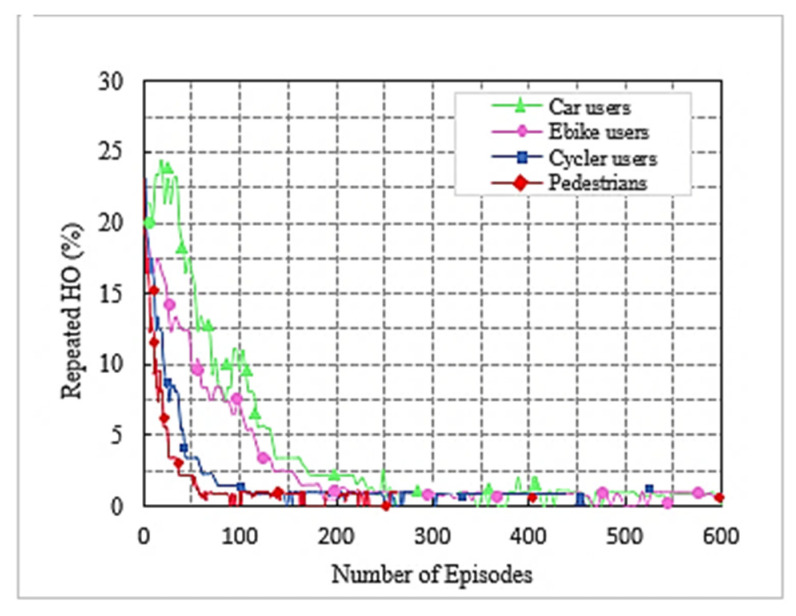
Number of wasteful HO as a function of the number of training episodes for JMLS–DDPG HO scheme.

**Figure 7:**
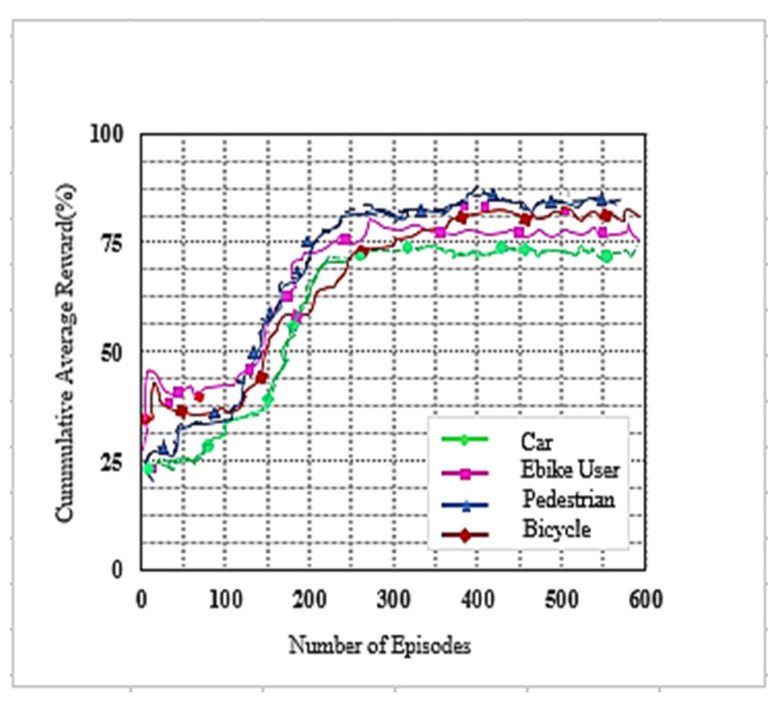
The cumulative reward as a function of the number of training episodes for DDPG only HO scheme according to user type.

**Figure 8:**
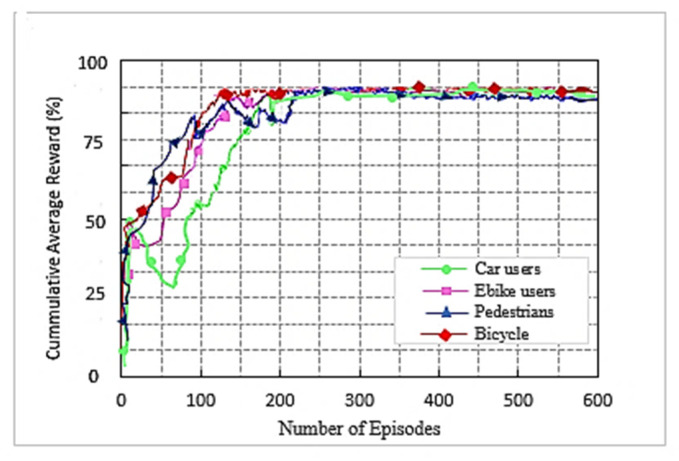
Cumulative reward as a function of the number of training episodes for our proposed JMLS–DDPG HO scheme according to user type.

We used the DC LTE mmWave model introduced by the NYU and the University of Padova in our simulation [1]. The LTE BSs in the DC model manage mmWave BS. The model carefully considers the end-to-end mmWave cellular network performance. It uses an ns-3 simulator and features a 3GPP channel model for frequencies above 6 GHz, as well as a 3GPP-like cellular protocol stack [1]. The JMLS–DRL algorithm was developed using the OpenAI Gym [24] toolkit. Open AI Gym is an RL development that is integrable with the ns-3 simulator; it supports teaching agents for a variety of network applications including those in ns-3. We investigated the performance using system-level simulations. Data collected from over 1000 s of simulation time with a resolution of one transmission time interval (TTI) (1 ms) were used for analysis. The main parameters used are summarized in Table 2. For a more detailed review of simulators, refer to [15]. Figure 5 and Figure 6 compare the number of wasteful HOs as a function of the number of training episodes in the DRL HO scheme and JMLS–DDPG HO scheme, respectively. The former gets new training samples from the environment once the initial pattern has been defined by EM estimations for every other episode, while the latter uses EM estimated data as the training sample as long as condition (5c) is satisfied. It only requests new training samples when EM data estimates fail to meet condition (5c). Results show that our proposed scheme quickly reduces the number of wasted HOs compared to the DDPG only HO scheme. For instance, it required 250 episodes to reduce repeated HOs to minimal levels of less than five, whilst the DDPG only scheme required close to 400 episodes. This also suggests that it can strategically and ably predict deterioration patterns using fewer training samples. The fact that this is more reliable and accurate than a method that continuously receives new training samples was justified in [4]. The authors in [4] argued that the angles of arrival and received power slowly vary with speeds because they are affected by the large-scale scattering environment and do not change with small-scale mobility. Since the received power samples do not change significantly from one sample to the next, we can use the training samples of the received power in meta training. Figure 7 and Figure 8 compare the cumulative average reward behavior as a function of training episodes under different user types. We can draw several observations. First, the early predictions or rewards of the deterioration pattern for different user types are very fuzzy in the JMLS–DDPG scheme. This explains why there are a high number of wasteful or repeated HOs in the early part of the training of JMLS–DRL, as shown in Figure 6.

**Figure 9:**
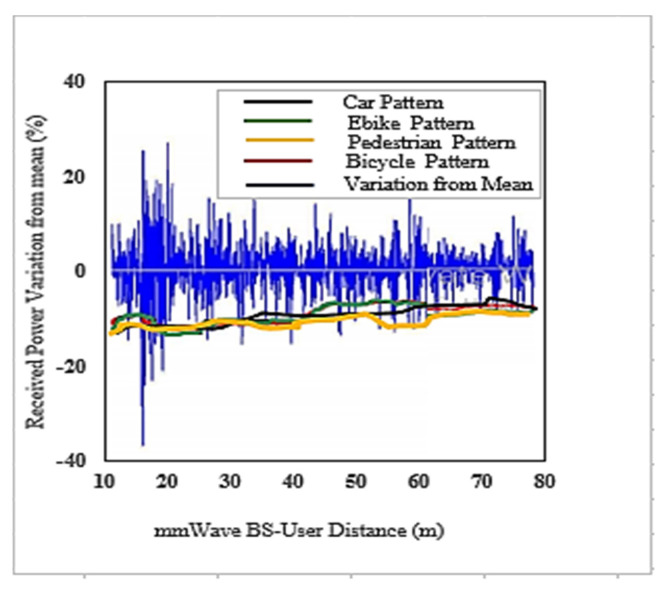
The actual average received power pattern variation at the UE as a percentage about the mean value after 200 episodes in the proposed JMLS–DDPG HO scheme.

**Figure 10:**
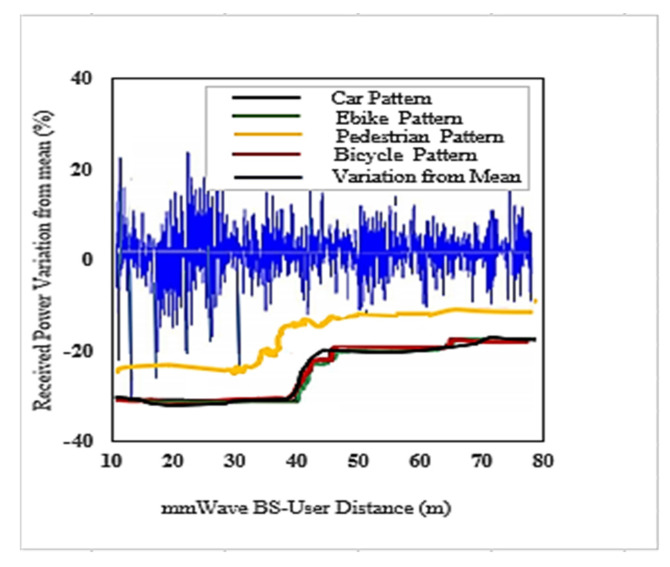
Best expected received power pattern variation as a percentage about the mean value after 500 episodes over 80 m in the proposed JMLS–DDPG HO scheme.

**Figure 11:**
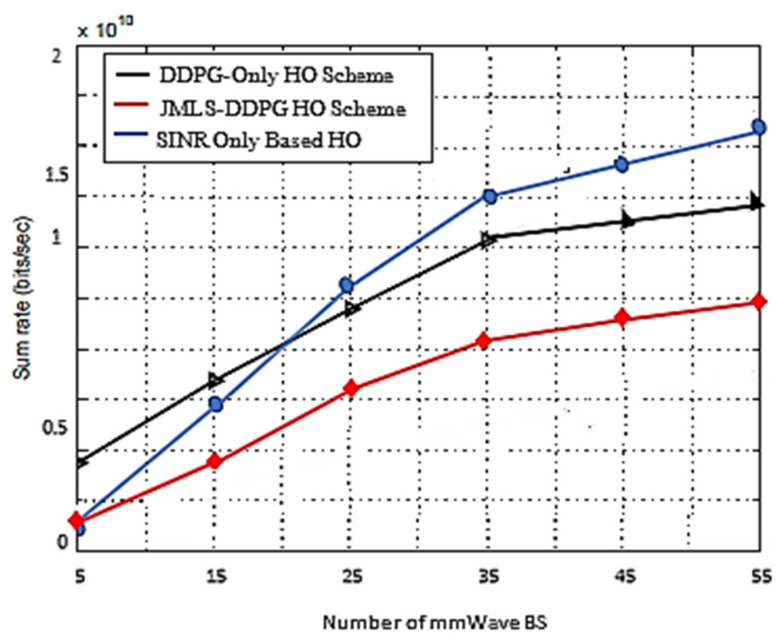
Sum rate for three HO schemes as a function of the number of BSs.

**Figure 12:**
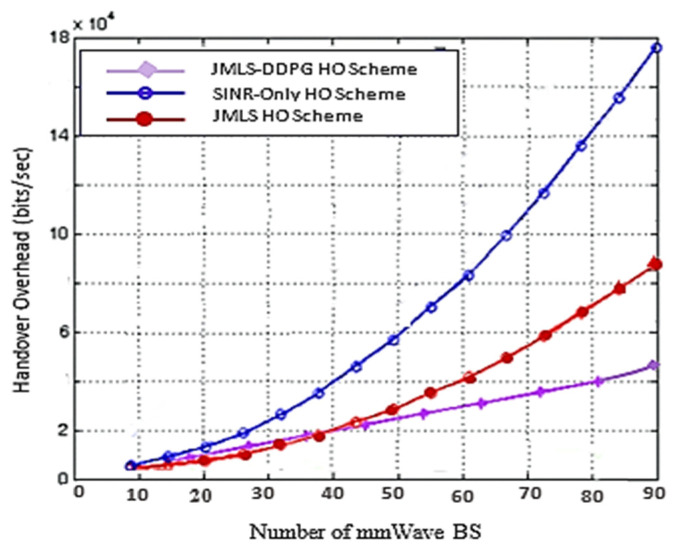
Overhead as a function of the number of mmWave BSs.

The blurriness is also seen when we compare the deterioration pattern prediction after 200 episodes in Figure 9 and after 500 episodes in Figure 10. Figure 10 shows a more accurate prediction of likely received power for different user types than Figure 9 with 200 episodes or observations in our proposed JMLS–DRL-empowered HO algorithm. Secondly, while the DDPG scheme converges independently for each user type as seen in Figure 7, the proposed JMLS–DRL scheme converges with almost a common and higher reward for all user types (see Figure 8). The implication is that, after 200 training episodes, the JMLS–DRL algorithm can have one common/global deterioration pattern to follow regardless of user type. On the other hand, for the DDPG HO scheme, each user type will need to follow a different type of deterioration pattern. This facilitates our proposed scheme’s prediction of the expected target link behavior. In both schemes, an HO is only issued when the received power at a particular given state/distance from the serving BS drops beyond the corresponding value of the expected local deterioration pattern. In this case, the global and local deterioration patterns in KMO are compared at least within a range of 80 m from a serving mmWave BS. While we can still try and predict beyond 80 m, the computation cost will be too high. Thus, a selected target link is deemed reliable if it is able to sustain connectivity within the 80 m transmission range. Beyond 80 m, HOs are evoked if the SINR drops to at least within 3 dB of the threshold. Therefore, HOs select a link on the basis of the fact that sustained connectivity is expected for at least for 80 m of assumed coverage of the mmWave BS. We also analyzed a soft-HO DC-based scheme [4] using only SINR [2] and a DDPG-based scheme [3] for comparison; the former acted as a baseline for our case in Figure 11 and Figure 12.

In Figure 11, we compare the sum rate as a function of the number of BSs for three different HO schemes. The SINR-based scheme, as explained in [4], only compares the SINR of the target and serving cell/link. The other scheme gets new updates every episode, whilst our proposed scheme uses both new and old CSI. We can see that the proposed scheme has good efficiency in terms of how it uses/selects BSs. The other two schemes seem to start saturating after 35–40 BSs. This can be attributed to the low training sample requirement and thorough analysis of CSI in our scheme. The reuse of training samples gives our scheme ample time to analyze the behavior of links. At the same time, having a small number of mmWave BSs prevents the proposed scheme from learning more about the target link deterioration pattern. This can be seen by the smaller sum date rate recorded at 5 to 15 mmWave BS. More mmWave BSs diversify the amount of data looked at in each episode. On the other hand, despite a very small number of BSs, for the DDPG only HO scheme, the acquisition of new training samples in each episode improved the prediction of the target link path; however, because it changed quickly, the inaccuracy in the predictions quickly manifested.

Another criterion to evaluate the performance of the proposed HO methods is the generated overhead. Figure 12 shows the variation of the induced overhead for the three proposed HO methods. It is obvious that the SINR-based HO induces more handover since, at each attachment to a new BS, a number of new measurement reports must be exchanged to allocate new subcarrier resources. On the other hand, using the DDPG only handover and our proposed HO scheme, fewer overheads are experienced because the past link data needed to achieve reliability are reusable and exchanged in advance before the HO. For our proposed scheme, this advantage is more evident because measurement data sources can be switched depending on condition (5c) (see Figure 3). Hence, the proposed scheme is better than both the DDPG only and the SINR HO schemes.

## 5. Conclusions and Future Works

This paper proposed a new HO scheme given the distinct propagation characteristics of mmWaves in a HetNet structure. A resource allocation problem that considers the utilization of mmWave bands with LTE bands in a multiuser setup was considered. We considered a downlink LTE-mmWave HetNet scenario with an mmWave link behavior pattern analysis scheme applied to address the HO challenges. The resulting optimization solution consisted of modeling the link behavior using JMLS, DRL, and meta training techniques. Subsequently, the optimal HO link was selected using KMO test principles. Simulation results showed that our HO scheme outperformed the DDPG only HO scheme and the SINR-only based HO scheme in terms of the number of successful HOs. Additionally, the proposed scheme had fewer wasted (repeated) HOs and a quicker reduction in repeated HOs. In particular, as plotted in Figure 5 and Figure 6, if we compare the number of repeated (wasted) HOs when using the existing DDPG (DRL) model and when using with our proposed JMLS–DRL scheme, results show that our scheme’s performance was better. For instance, within 200 training episodes, our scheme was able to reduce the total percentage of wasted HOs to less than 5%. This is unlike the DDPG only HO scheme that exhibited over 5% after 200 training episodes. In addition, we compared our proposed scheme with the DRL and SINR HO schemes in terms of the sum rate and overhead performance (Figure 10 and Figure 11, respectively). Our scheme also showed better performance in this regard. For instance, with a network of 55 mmWave BSs, the JMLS–DDPG HO scheme network had a sum rate of nearly 2 × 10^10^ bits/s and a corresponding overhead of less than 4 × 10^4^ bits/s, as shown in Figure 10 and Figure 11, respectively. This is unlike the DDPG only HO scheme which had a sum rate of less than 1.5 × 10^10^ bits/s and almost double the overhead (8 × 10^4^ bits/s) for the same number of mmWave BSs. Thus, we can conclusively state that the proposed HO scheme offers longer dew times (time between HOs) than the SINR-based and DDPG only HO schemes. The results demonstrate the vital role that deterioration pattern analysis can play in addressing mmWave link selection in 5G networks. Principally, we can conclude that our pattern analysis HO scheme envisages traits of long-term behavior analysis for mmWave target links before HO execution. This is unlike unreliable classic HO schemes (e.g., the SINR-based HO) where only the instantaneous behavior of target links is analyzed prior to choosing the best target link. In future work, it would be interesting to consider the competing effects of path loss, channel gain, and transmission power when determining the receivable deterioration pattern of the target link. This is given the impact that their variation has on the data rates. Furthermore, while there is a need for highly directional beam antennas at the PHY layer to have an acceptable link quality, how to effectively handle or dodge adverse effects of both mobile and static blockages when choosing mmWave links in HO schemes could be interesting to study in future behavior pattern projections studies for target links. Pattern analysis can also be extended to cell planning, coverage, or rate maximization. This is vital considering the vulnerability of mmWave to topographic and user dynamics. Lastly, studying backhaul configurations that can efficiently support the proposed HO scheme would also be interesting using the pattern-based HO scheme proposed.

## Figures and Tables

**Table 1:** Interpretation Of KMO Measure.

KMO	Interpretation
0.9 and above	Marvelous
0.8–0.9	Meritorious
0.7–0.8	Middling
0.6–0.7	Mediocre
0.5–0.6	Miserable
Under 0.5	Unacceptable

**Table 2:** Simulation Parameter Table [1].

Parameter	Value
mmWave	28 GHz
mmWave bandwidth	1 GHz
3GPP Channel Scenario	Urban Micro, Urban Macro
MMWave max outage	−5 dB
mmWave transmission Power	46 dBm
mmWave max PHY Rate	3.2 Gbps
X2 link latency	1 ms
S1 link latency	10 ms
RLC buffer Size	5 MB
S1 MME link latency	10 ms
User speed	[1,50] m/s

## Data Availability

Not applicable.
